# Effects of low dose intravenous sodium nitrite on arterial oxygenation and hemodynamics in experimental acute respiratory distress syndrome (ARDS)

**DOI:** 10.1186/2197-425X-3-S1-A574

**Published:** 2015-10-01

**Authors:** S Kronfeldt, P Lother, RCE Francis, T Busch, W Boemke, PA Pickerodt, ER Swenson

**Affiliations:** Department of Anesthesiology and Intensive Care Medicine, Campus Charité Mitte and Campus Virchow-Klinikum, Charité - Universitätsmedizin Berlin, Berlin, Germany; Department of Anesthesiology and Intensive Care Medicine, Universität Leipzig, Leipzig, Germany; Department of Pulmonary and Critical Care Medicine, University of Washington, Seattle, WA USA; Veterans Affairs Puget Sound Health Care System, Seattle, WA USA

## Introduction

Nitrite (NO_2_^-^) is an endogenous storage pool for nitric oxide (NO) [1]. We showed that sodium nitrite (NaNO_2_) mitigates ventilator-induced lung injury via NO dependent mechanisms in rats [2].

## Objectives

We hypothesized that low dose intravenous (i.v.) NaNO_2_ may improve arterial oxygenation and reduce mean pulmonary artery pressure (MPAP) and pulmonary vascular resistance (PVR) in ARDS in pigs.

## Methods

ARDS was induced in 12 pigs by surfactant depletion due to saline lung lavages [3]. Two groups were investigated for 5 h: 1. Controls (n = 6) and 2. NaNO_2_ i.v. (0.3 mg/kg BW bolus, followed by 0.1725 mg/kg BW continuously; n = 6). We measured mean arterial pressure (MAP), MPAP and cardiac output as well as exhaled NO (NOex), blood gases and Wet/Dry-Ratios of lung tissue.

## Results

At baseline the arterial oxygen tension (P_a_O_2_) was 539 ± 50 mmHg and 508 ± 35 mmHg in Controls and NaNO_2_ i.v. respectively (fraction of inspired oxygen = 1.0). P_a_O_2_ decreased to 67 ± 17 mmHg (Controls) and 57 ± 13 mmHg (NaNO_2_ i.v.) after ARDS induction. During the protocol, P_a_O_2_ increased to 120 ± 73 mmHg (Controls) and 103 ± 82 mmHg (NaNO_2_ i.v.). NOex was unchanged in both groups. Lung Wet/Dry-Ratios were 8.1 ± 0.8 (Controls) and 8.9 ± 0.7 (NaNO_2_ i.v.). For hemodynamic values see Table 1 (all values: mean ± SD).

**Table 1:** Table 1

Groups	TP	MPAP (mmHg)	PVR (dyn*s*cm-5)	MAP (mmHg)	SVR (dyn*s*cm-5)	CO (L/min)
Controls	T0	14 ± 3	205 ± 107	92 ± 6	2157 ± 520	3.5 ± 0.9
	T1	33 ± 6	360 ± 97	74 ± 13	966 ± 212	6.2 ± 0.7
	T2	27 ± 5	243 ± 78	67 ± 10	798 ± 196	6.6 ± 0.9
NaNO2 i.v. low dose	T0	15 ± 1	185 ± 26	90 ± 13	1967 ± 417	3.7 ± 0.4
	T1	34 ± 3	429 ± 78	72 ± 10	1092 ± 293	5.1 ± 0.5
	T2	29 ± 6	284 ± 82	68 ± 11	816 ± 320	6.9 ± 1.6
Mean pulmonary artery pressure (MPAP), pulmonary vascular resistance (PVR), mean arterial pressure (MAP), systemic vascular resistance (SVR), cardiac output (CO) in control animals (Controls; n = 6) and in animals treated with low dose intravenous sodium nitrite (NaNO2 i.v.; n = 6); Time point of measurement (TP): Baseline (T0); ARDS baseline (T1); End of experiment (T2); All values: mean ± SD						

## Conclusions

Lung lavage induced severe ARDS with increased MPAP and PVR in both groups. I.v. application of low dose NaNO_2_ did not reduce lung edema formation and did not improve arterial oxygenation or pulmonary hemodynamics in this model of severe ARDS in pigs.

## Grant Acknowledgment

This study was supported by the Deutsche Forschungsgemeinschaft (PI795/2-1).
